# Perceptual Learning of Time-Compressed Speech: More than Rapid Adaptation

**DOI:** 10.1371/journal.pone.0047099

**Published:** 2012-10-09

**Authors:** Karen Banai, Yizhar Lavner

**Affiliations:** 1 Department of Communication Sciences and Disorders, University of Haifa, Haifa, Israel; 2 Department of Computer Science, Tel-Hai College, Israel, Tel-Hai, Israel; University of Salamanca- Institute for Neuroscience of Castille and Leon and Medical School, Spain

## Abstract

**Background:**

Time-compressed speech, a form of rapidly presented speech, is harder to comprehend than natural speech, especially for non-native speakers. Although it is possible to adapt to time-compressed speech after a brief exposure, it is not known whether additional perceptual learning occurs with further practice. Here, we ask whether multiday training on time-compressed speech yields more learning than that observed during the initial adaptation phase and whether the pattern of generalization following successful learning is different than that observed with initial adaptation only.

**Methodology/Principal Findings:**

Two groups of non-native Hebrew speakers were tested on five different conditions of time-compressed speech identification in two assessments conducted 10–14 days apart. Between those assessments, one group of listeners received five practice sessions on one of the time-compressed conditions. Between the two assessments, trained listeners improved significantly more than untrained listeners on the trained condition. Furthermore, the trained group generalized its learning to two untrained conditions in which different talkers presented the trained speech materials. In addition, when the performance of the non-native speakers was compared to that of a group of naïve native Hebrew speakers, performance of the trained group was equivalent to that of the native speakers on all conditions on which learning occurred, whereas performance of the untrained non-native listeners was substantially poorer.

**Conclusions/Significance:**

Multiday training on time-compressed speech results in significantly more perceptual learning than brief adaptation. Compared to previous studies of adaptation, the training induced learning is more stimulus specific. Taken together, the perceptual learning of time-compressed speech appears to progress from an initial, rapid adaptation phase to a subsequent prolonged and more stimulus specific phase. These findings are consistent with the predictions of the Reverse Hierarchy Theory of perceptual learning and suggest constraints on the use of perceptual-learning regimens during second language acquisition.

## Introduction

Rapidly delivered speech is harder to comprehend than slower rate speech, and even more so for non-native speakers [1], [2], older adults and individuals with hearing impairment [3]. Although the ability to identify time-compressed speech, an artificial form of fast speech, improves rapidly over the course of listening to as few as 10–20 sentences [4], [5], [6], [7], [8], [9], the characteristics of learning and generalization beyond this initial adaptation phase have not been fully described. In fact, it is not even clear if practice beyond the adaptation phase yields additional learning because most studies on the perceptual learning of rapid speech focused on the adaptation period. Moreover, even experienced non-native listeners benefit from slower than normal speech rates [2], [10]. These findings suggest that under ecological conditions, even prolonged and intensive experience does not result in native-like performance and can be taken to indicate that long-term perceptual learning of rapid speech is limited. As for generalization, the continued difficulties of non-native speakers suggest that long-term learning associated with prolonged experience might be quite specific, in contrast to the generalization across tokens and speech materials associated with brief adaptation to rapid speech. The goal of the present investigation was therefore to determine whether multiday practice on the verification of sentences presented in a time-compressed format in a group of non-native Hebrew speakers, is more beneficial than a brief exposure period. Another goal was to test the profile of generalization following multiday perceptual learning (if such learning occurs) To those ends, highly fluent non-native Hebrew speakers were trained on the verification of sentences presented in time-compressed form for five sessions. During each session they had to verify 300 sentences. Before and after training their performance on the trained condition and four additional conditions designed to assess the generalization of learning was compared to that of untrained listeners who participated in the pre- and post-test sessions only.

The identification of time-compressed sentences improves rapidly with repeated exposure. In previous studies [4], [5], [6], [7], [8], [9], listeners were presented with sentences compressed to a predetermined fraction (30–45%) of their original duration. Initially, listeners were able to reconstruct only part (20–76%) of the words in any given sentence. Brief adaptation (10–20 sentences) significantly improved identification, but left performance well below perfect (40–85% correct). These findings suggest that further learning could occur with more practice. Although we are aware of no studies in which the effects of multi-session practice on time-compressed speech were reported, studies with other speech tasks suggest that further learning often occurs with longer training. For example, both speech in noise perception [11] and phoneme discrimination [12], [13], [14],[15] were found to improve with multi-session training. Furthermore, when listeners were adapted to 20 time-compressed sentences on one session and brought back to the lab a week later, further improvements were observed during a second adaptation session, even though some of the improvement was retained between sessions [8]. It was hard to determine whether this additional improvement reflected re-adaptation (to the level observed at the end of the initial adaptation phase) or further learning, but either way, this finding suggests that learning of time-compressed speech might benefit from longer training. Therefore, we now hypothesize that multi-session practice (5 sessions of 300 sentences each) will result in additional performance gains to those induced by participation in a pre- and post-test sessions only.

Brief adaptation to time-compressed speech improves not only the identification of the trained materials, but also the ability to identify rapid speech presented either by different talkers, in a different language and at different compression rates. For example, after adapting to 10 sentences presented by a single talker at a particular compression rate, the performance of adapted listeners with novel materials presented by a new talker or at a new compression rate was better than that of naïve listeners who did not previously adapt to time-compressed speech [7]. Similarly there is evidence that brief training with (artificially) time-compressed speech generalizes to natural fast speech [6] and even across languages that share similar phonetic structures (e.g., Spanish and Catalan) [4], [9]. This pattern of generalization was interpreted to suggest that adaptation to time-compressed speech involves a phonological, rather than an acoustic or lexical level of processing. At this level, the representations of speech are acoustically invariant, compatible with the transfer across compression rates. Furthermore, representations are not limited by the lexical status of the materials used, explaining the transfer across phonetically similar languages even when listeners did not speak both languages [4], [9], or after adaptation to sentences comprised of nonsense words [16]. As for long-term learning, two possibilities arise. If long-term learning of time compressed speech is simply a continuation of the rapid adaptation phase, it is reasonable to expect qualitative similarities between the outcomes of rapid adaptation and multi-session practice. For example, one could surmise that broad generalization to untrained materials will follow multi-session practice. On the other hand, if rapid adaptation and prolonged practice are qualitatively distinct, the pattern of generalization is expected to differ between the two phases. A theoretical framework in which different patterns of generalization characterize different phases of learning is proposed by the Reverse Hierarchy Theory (RHT) of perceptual learning [17], explained below.

The RHT suggests that although the neural processing of sensory information proceeds in a bottom-up manner, creating increasingly abstract representations of the physical stimulus, conscious perception operates in a top-down manner. Our initial perception of an acoustic event is thus based on higher-level abstract representations which are accessible to naïve listeners [18], [19]. By this account, limitations to naïve performance on tasks that rely on the use of perceptual information arise due to the initial inaccessibility of the relevant representations rather than due to a lack of sufficiently detailed sensory representations of the relevant stimulus dimensions [17], [20]. Naïve performance on time-compressed speech tasks is not poor due to inherent difficulties in encoding rapid speech, but rather because initial performance relies on high-level abstract acoustic representations and not on detailed low-level spectro-temporal representations of speech. In this theoretical framework, perceptual learning, like perception, is a top-down driven process intended to locate the most relevant sensory representations. Thus, whereas naïve performance is based on high-level abstract representations, the performance of highly-trained individuals is based on the information contained in fine-grained low-level representations. Because low-level representations are more stimulus specific, with more practice, learning is expected to become more and more specific [20]. The top-down search process is slow and effortful. Therefore, low-level representations should become accessible only under specific training [19], [21].

The RHT has been formulated based on studies in the visual modalities, and was extended to the auditory modality only recently [17], [19]. Those recent studies provide support for RHT predictions regarding the accessibility of higher and lower level representations, but the idea that with continued practice learning should become more specific was not directly tested. Nevertheless, data other from studies on the perceptual learning of acoustic discrimination are generally consistent with the RHT claims in showing that intensive practice often yields learning that is quite specific to the trained stimuli (see [22] for a recent review). For example, multi-session practice on temporal-interval discrimination results in improvements that are specific to the practiced temporal interval (e.g., 100 ms), with no generalization to nearby untrained intervals (e.g., 50 and 200 ms) [23]. Likewise, following intensive training on speech tasks, little generalization to untrained contexts has been reported [24], [25]. The pattern of generalization following brief adaptation to time-compressed speech is also consistent with the predictions of the RHT. Brief adaptation is not sufficient to engage detailed low level acoustic representations, but it might be sufficient to activate phonological representations [4]. In contrast, prolonged practice is expected to initiate the top-down search process required to engage more detailed lower-level representations. Because those representations are by nature more specific, prolonged training is expected to result in less generalization than brief training. Therefore, we hypothesize that multi-day practice on a time-compressed speech task will result in additional perceptual learning to that reported after a brief adaptation phase, but this learning will be more stimulus specific and not generalize as broadly.

## Methods

### Listeners

A total of 64 University of Haifa undergraduate students (aged 18–28) participated in the study. Participants were naïve to psychophysical testing and by self report had no known speech, hearing or learning problems. Participants were compensated for the time devoted to the study. All aspects of the study were approved by the ethics committee of the Faculty of Social Welfare and Health Sciences at the University of Haifa.

Participants included 44 native Arabic speakers, and a comparison group of 20 native Hebrew speakers. Arabic speakers were highly proficient Arabic/Hebrew bilinguals using both languages on a daily basis. By self report, Hebrew speakers did not speak or understand Arabic to any meaningful degree. Although they demonstrated sufficient fluency in English to earn university entrance, they were not using English or any other language daily and have always attended Hebrew speaking schools.

The participants were divided into 3 groups as follows: (1) The trained group, comprised of 20 Arabic speakers who participated in the training program; (2) The control group, comprised of 24 Arabic speakers who did not train but participated in pre- and post-tests only; (3) The native comparison group comprised of 20 native Hebrew speakers tested once to obtain baseline estimates of performance on our training and generalization tasks. See below for further details on the training program and testing procedure.

### Organization of the Experiment

The experiment had three phases, a pre-test taken by all participants, a training phase completed by the trained group, and a post-test completed by the trained group as well as the control group. The pre- and post-test sessions, conducted 10–14 days apart included five different conditions of time-compressed speech verification (see below). In between the pre- and post-test sessions, the trained listeners practiced on one of the speech verification conditions (see below). The control group received no training.

### Tasks and Adaptive Procedure

A sentence verification task and a lexical decision task were used. In the sentence task listeners heard a sentence and had to determine whether it was semantically correct or not. In the lexical decision task they had to decide whether each stimulus was a real Hebrew word. Half the stimuli in each condition were true (semantically correct sentences or real words) and half were false (semantically incorrect sentences or non-sense words). Stimuli were presented binaurally over headphones in blocks of 60 (during the training phase) or 80 (during the pre- and post-test phases) trials using an interactive computer program which administered the adaptive staircase procedure and recorded the listener responses. Listeners had to respond within 5 seconds of stimulus presentation by selecting one of two on-screen pushbuttons (‘true’, ‘false’) and received visual feedback after each response (a smiley face following correct responses and a sad-smiley face following incorrect responses). No response within the 5 seconds of the response window was considered as incorrect. The order of the sentences within a block of trials was selected at random (without replacement).

In both the pre/post tests, as well as in the training sessions, a modified up-down staircase procedure was used [26], [27] to adjust the level of time-compression based on the performance of each participant. A two-down one-up procedure was employed, with an initial compression level of 65%. Compression level was modified logarithmically, using a scale of 25 logarithmically equal steps between the starting value and the maximal level of compression (20%). Trials on which changes in the direction of the function relating trial number and compression level occurred were labeled reversals.

Time compression throughout the experiment was carried out using an implementation of the WSOLA (Waveform Similarity Overlap and Add) algorithm [28], which has been shown to achieve very high quality time-scale modification of speech signals [29]. Like other OLA-based algorithms [30], WSOLA modifies the rate at which the speech signal is presented, while maintaining other qualities, such as the pitch and the timbre, unchanged.

### Stimuli

All stimuli were recorded and sampled at 44 kHz using a standard microphone and PC soundcard by a young male native speaker of Hebrew (the trained speaker). In addition, a subset of the sentences (the training list) were recorded by two more native Hebrew speakers (one male and one female) and were used for tests of across-talker generalization during the pre- and post-test phases.

#### Sentences

A total of 200 simple active subject-verb-object (SVO) sentences in Hebrew were used in this study, following Prior and Bentin [31]. Each sentence was 5–6 words long and had adjectives modifying both the subject and the object. The naturally spoken sentences had an average duration of 3 seconds (range: 2.3–4.2 s) and an average rate of 109 words/minute (range 72–144). One hundred sentences were semantically plausible (true, e.g., “The municipal museum purchased the impressionistic painting”) whereas the remaining sentences (false) had a semantic violation in either the subject or the verb position which made them implausible (e.g., “The eloquent speaker recited the impressionistic painting”). The pool of sentences was divided to a training list (n = 100 sentences, 50 of which were ‘true’) and a generalization list. Training and generalization lists were the same for all participants.

#### Single words and pseudo-words

104 two syllable stimuli (52 common Hebrew words and 52 pseudo-words) were used during the pre- and post-test phases of the study. Each pseudo-word was a minimal pair of one of the real words (e.g., the real word ‘Ma*f*sek’ (a switch in Hebrew) and its counterpart ‘Ma*k*sek’). The phoneme distinguishing the word/pseudo-word pairs was always a consonant and could occur in an initial, middle or final position within the word.

### Pre- and Post-test Conditions

Five different conditions were administered to each listener in the pre-test session, the first session in which both groups of listeners participated. The same five conditions were administered in the post-test session, which was conducted about 10–14 days after the pre-test session. In four of the conditions, a sentence verification task was used; in the remaining condition a lexical decision task was administered as follows, with the order of conditions counterbalanced across listeners.

1. The trained condition. 80 sentences (half of them true) were selected at random (without replacement) from the training set and presented by a male speaker (designated the trained speaker).
2. Across-token generalization condition (untrained tokens). 80 sentences (half of them true) were selected at random from the generalization set. Those were presented by the same talker as the trained condition.
3. Across-talker generalization – male. A different male talker was used to present 80 sentences (half of them true) selected at random (without replacement) from the training set
4. Across-talker generalization – female. Same as condition 3, but with a female speaker.
5. Sentence-to-word generalization. 80 single word/pseudo-word stimuli selected at random from the pool of 104 stimuli, presented by the trained speaker.

### The Training Regimen

Five sessions were administered over the course of 10 days. During each session, listeners had to verify sentences taken from the training set presented in 5 adaptive blocks of 60 trials. On average, 30–40 minutes were required to complete each training session (including brief breaks between blocks if needed).

### Data Analysis

Two indices of individual performance were used: thresholds and performance consistency. Threshold was calculated as the mean compression level across the last 5 reversals on each block. Performance consistency was defined as the standard deviation of the same reversal values used for threshold calculation, providing an indication of how stable the mean threshold is.

Learning was defined as significantly larger pre-to-post-test gains (in thresholds and performance-consistency) in trained versus control listeners. Statistically, this was determined using two time (pre- vs. post- test) X two group (trained vs. control) analysis of variance (ANOVAs), with time as a repeated measure, conducted on the data of the trained condition. The ANOVA was followed by two planned t tests comparing (1) the pre-test thresholds, and (2) the magnitude of the pre- to post-test changes between trained and control listeners. Likewise, generalization was determined with similar ANOVAs and planned t-tests conducted on each of the generalization conditions. A significant interaction term accompanied by lack of pre-test differences between the groups and greater pre- to post-test changes in the trained group than in the control group were taken as evidence of significant learning/generalization. Because only two independent planned comparisons are possible with our design, no comparison was directly performed on post-test values.

## Results

### Group Effects

**Figure 1:**
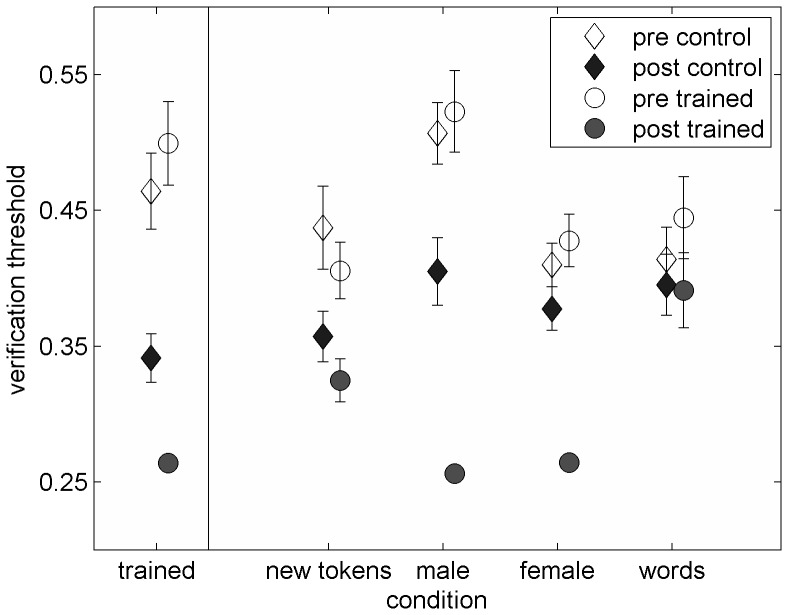
Pre- and post-test average verification thresholds. From left to right thresholds are shown for the trained condition and four conditions designed to test generalization to untrained sentences (new tokens), untrained speakers (different male and different female) and to non-sentence materials (single words). Controls are marked with diamonds; trained listeners are marked with circles. Thresholds are expressed in fraction of original sentence duration, thus a value of 0.5 is equivalent to a sentence presented at twice its original rate. Pre-test thresholds are marked with empty symbols, post-tests with filled symbols. Error bars are ±1 standard error of the mean.

Mean pre- and post-test thresholds of the trained and the control group are shown in Figures 1 (average thresholds) and 2 (performance consistency). Statistically, 2 by 2 ANOVAs with group as a between-subject factor and time as a within-subject factor reveal that average performance on the trained condition was similar between the two groups (F(1,42) = 0.70, p = 0.41). Likewise, there were no group effects for the average thresholds on the untrained-tokens condition (F(1,42) = 1.23, p = 0.27) and on the sentences-to-words generalization condition (F(1,42) = 0.01, p = 0.98). Significant group effects were found on the two talker-generalization conditions (Male: F(1,42) = 5.36, p = 0.025; Female: F(1,42) = 4.37, p = 0.04). As for performance consistency, there were no group effects for any of the conditions except for the untrained-male condition (F = 5.44, p = 0.025).

**Figure 2:**
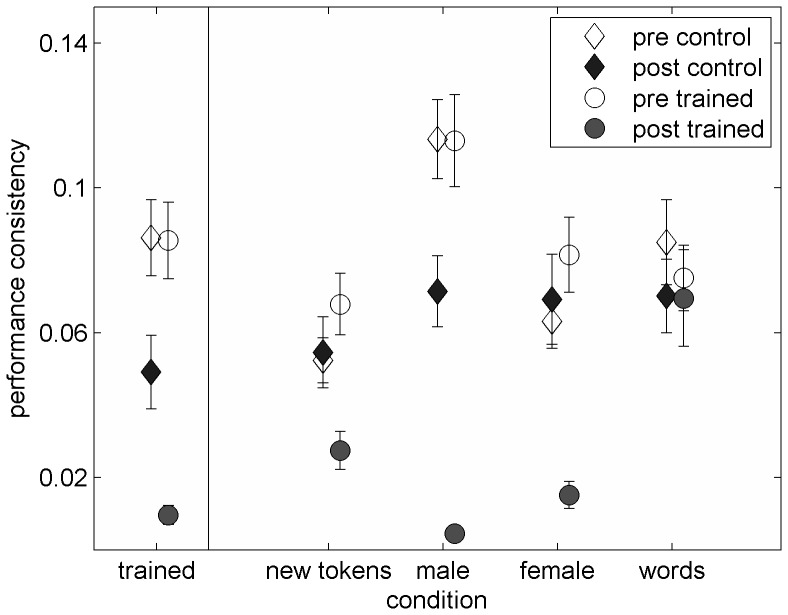
Pre- and post-test performance consistency. Consistency was estimated as the standard deviation across all threshold estimates observed for each individual subject for each condition. See caption of Figure 1 for further details.

A visual inspection of Figures 1 and 2 suggests that pre-test thresholds and performance consistency were comparable between the two groups. As shown in Table 1, planned comparisons support those observations for all conditions and both indices of performance. Taken together, these data suggest that the two groups were similar on the test conditions at the onset of the study. Therefore, following analyses concentrate on the effects of time and training.

**Table 1:** Pre-test performance – planned comparisons between the trained and the control groups.

	Average performance			Performance consistency		
Condition	Contrast Value	t(42)	p	Contrast Value	t(42)	p
Trained	0.04	0.68	0.50	−0.0008	−0.04	0.97
Untrained Tokens	−0.03	−0.65	0.52	0.02	1.05	0.30
Different Male	0.01	0.15	0.88	−0.0004	−0.02	0.99
Different Female	0.02	0.59	0.57	0.02	1.03	0.31
Single Words	0.1	0.30	0.76	−0.01	−0.46	0.64

### Pre- to Post-test Changes

Figure 1 suggests that verification thresholds in both groups of listeners improved between the pre- and the post-test assessments in the trained condition, as well as in three of the four untrained conditions. 2 by 2 ANOVAs with assessment (pre, post) as a within-subject factor and group as a between-subject factor suggest those changes were significant for the trained condition (F(1,42) = 47.95, p<0.001) as well as in the untrained tokens condition (F(1,42) = 10.21, p = 0.003), the untrained male condition (F(1,42) = 40.70, p<0.001) and the untrained female condition (F(1,42) = 19.82, p<0.001), but not in the single-word condition (F(1,42) = 0.86, p = 0.36).

Figure 2 similarly suggests that performance consistency improved in both groups between the two assessments on the trained condition as well as on the generalization conditions with untrained speakers. Statistically, significant effects of time were found on the trained condition (F(1,42) = 23.88, p<0.001), the untrained-tokens condition (F(10,42 = 10.21, p = 0.003), the untrained-male condition (F(1,42) = 31.44, p<0.001) and the untrained-female condition (F(1,42) = 5.40, p = 0.025), but not on the single-words condition (F(1,42) = 0.52, p = 0.48). Therefore, to determine whether multi-day practice had additional effects to those induced by participation in the pre- and post-test sessions only, the time by group interaction terms and pre- to post-test difference scores will be explored in the next section.

### Learning and Generalization in the Trained Group Relative to the Control Group

Multiday training on rapid-speech verification resulted in additional learning and generalization to those induced by participation in the pre- and post-test assessments alone (see Figure 1). Statistically, additional learning and generalization were defined as the presence of a significant interaction term on a 2 by 2 ANOVA with time (pre-, post-test) as a within-listener factor and group (trained, control) as a between-listener factor. As shown in Figure 1, trained listeners learned significantly more than controls on the trained condition (Interaction F_(1,42)_ = 8.47, p = 0.006, partial η^2^ = 0.17). Furthermore, trained listeners significantly generalized their learning to the two untrained conditions in which different speakers presented the trained sentences (female: Interaction F_(1,42)_ = 13.62, p = 0.001, partial η^2^ = 0.25; male: Interaction F_(1,42)_ = 10.97, p = 0.002, partial η^2^ = 0.21). Although both groups of listeners significantly improved on the untrained condition with untrained sentences, no greater learning was observed in the trained than in the untrained group (Interaction F_(1,42)_ = 0.21, p>0.05). Likewise, practice did not induce any additional gains on the single word condition between the pre- and post-tests (Interaction F(1,42) = 0.11, p = 0.74). Planned comparisons on the difference scores between the pre- and the post-test confirm that in all the cases in which significant interaction terms were observed, trained listeners improved significantly more than untrained ones (see Table 2). Furthermore, although untrained listeners did improve on some of the conditions, effect sizes were always greater in the trained group (see Table 2).

**Table 2:** Pre- to post-test difference scores (± s.d), effect sizes and planned group comparisons on the difference scores.

	Trained Group		Control Group		Planned Between Groups Comparison	
	Difference Scores	Cohen’s D	Difference Scores	Cohen’s D	Contrast Value	t^1^
**Average performance**						
Trained	0.24±0.2	1.37	0.10±0.1	0.56	0.14	2.83**
Untrained Tokens	0.08±0.1	0.56	0.06±0.2	0.42	0.02	0.46
Different Male	0.28±0.2	1.32	0.09±0.2	0.42	0.19	3.31**
Different Female	0.17±0.1	1.08	0.02±0.1	0.10	0.15	3.69**
Single Words	0.04±0.2	0.21	0.01±0.2	0.07	0.03	0.44
**Performance Consistency**						
Trained	0.08±0.1	0.97	0.04±0.1	0.48	0.04	1.68*
Untrained Tokens	0.04±0.1	0.57	0.0±0.1	–	0.04	2.05*
Untrained Male	0.11±0.1	1.16	0.04±0.1	0.45	0.07	2.48**
Untrained Female	0.05±0.1	0.57	0.0±0.1	–	0.05	2.17*
Single Words	0.00±0.1	–	0.01±0.1	0.16	−0.009	0.33

1 the number of degrees of freedom was 42 for all between group comparisons except for the trained condition. For that condition, equal variances in the two groups could no longer be assumed after training and the degrees of freedom were adjusted accordingly to 33.7.

* p≤0.05.

** p<0.01.

*** p<0.001.

Similar to the training induced gains in average performance, individual performance consistency also improved with multiday practice (see Figure 2). This improvement was almost significant for the trained condition (Interaction F_(1,42)_ = 2.8, p = 0.1, partial η^2^ = 0.06). Furthermore, significant generalization was observed to the untrained condition with untrained sentences (Interaction F_(1,42)_ = 4.20, p = 0.047, partial η^2^ = 0.09) and to the two conditions with different speakers (Male: Interaction F_(1,42)_ = 6.14, p = 0.017, partial η^2^ = 0.13; Interaction Female: F_(1,42)_ = 7.80, p = 0.008, partial η^2^ = 0.16). When effect sizes of the interaction terms are considered (expressed here with partial η^2^) in addition to the ANOVA outcomes it seems that training induced improvements on performance consistency, were somewhat weaker than those observed for average performance. Nevertheless, multiday training resulted in additional improvements in performance consistency to those observed after participation in the pre- and post-tests only on four out of the five conditions, as shown in Table 2.

### Learning during the Training Phase in the Trained Group

**Figure 3:**
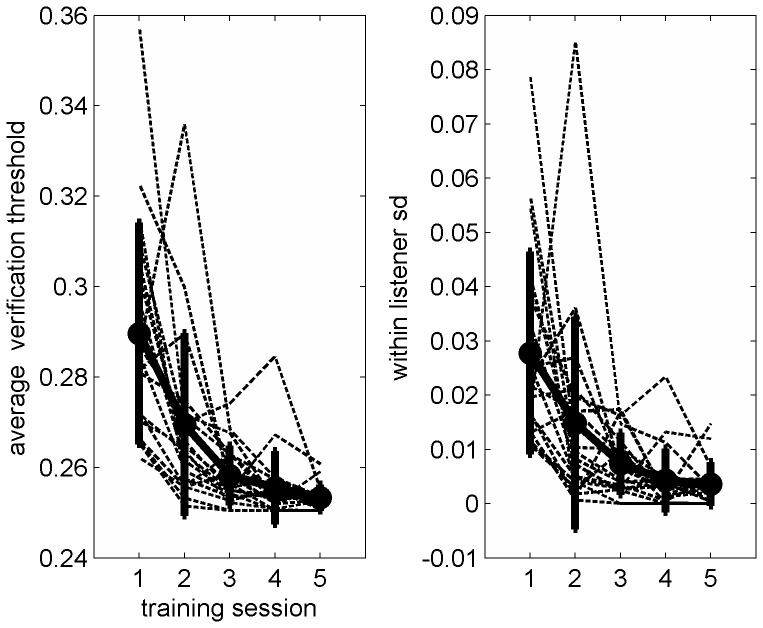
Learning curves. Left. Average verification thresholds. Right. Performance consistency. Individual listeners’ data is marked with dashed line. Group mean data is marked with a black line. Error bars are ±1 SD. The data of one listener with a mean session one threshold of 0.85 are not shown on the figure because they obscure the remaining learning curves. These data are included in all statistical analyses, but removing them had no influence on any of the outcomes.

Analysis of the learning curves from the training phase of the study further strengthens the conclusion that learning on the trained condition continued beyond the initial adaptation phase. As shown in Figure 3, both average daily thresholds (the mean thresholds across the 5 daily blocks) and daily performance consistency (the standard deviation across the 5 daily threshold values) gradually improved across the training sessions in 19/20 participants. For each participant, regression lines were fitted through the average threshold or consistency values from each practice session. For average thresholds, the slopes of all 20 participants were negative. The group mean slope of –0.0144±0.026 was significantly smaller than zero as suggested by a confidence interval in the negative range (95% confidence interval: −0.0266 to −0.0021). Likewise, for performance consistency, learning curve slopes were negative in 19 of 20 participants. The consistency group mean slope was −0.0051±0.005 also with a confidence interval in the negative range (95% confidence interval: −0.0075 to −0.0026). Therefore, the significant perceptual learning reported in comparison to the control group based on the pre- and post-test data is accompanied by significant improvements during the training phase.

To determine whether the rate of learning changed during the course of training, as suggested by Figure 3, repeated measures ANOVAs were conducted on daily threshold and consistency values followed by post-hoc t-tests between values in each two consecutive days. For average thresholds, thresholds improved significantly between the first two training sessions, with marginal improvements on the two subsequent sessions (F_(4,76)_ = 0.001, post hoc t-tests: day 1 vs. day 2: t_(19)_ = 3.48, p = 0.003; day 2 vs. day 3: t_(19)_ = 1.99, p = 0.065; day 3 vs. day 4: t_(19)_ = 1.96, p = 0.062). A similar trend was observed for performance consistency although it failed to reach significance (F_(4,76)_ = 1.53, p = 0.19). To summarize the changes in performance during the training period, we calculated the amount of change from the beginning to the end of training. Between the first and last training sessions, mean verification thresholds improved by 6±10% on average with an intermediate effect size (Cohen’s d = 0.56). The effect size of the change in performance consistency between the first and last practice sessions was large (Cohen’s d = 1.1). Taken together, these changes suggest that learning during the training phase was robust.

### Comparison of Trained and Untrained Arabic Speakers to Naïve Native Hebrew Speakers

**Figure 4:**
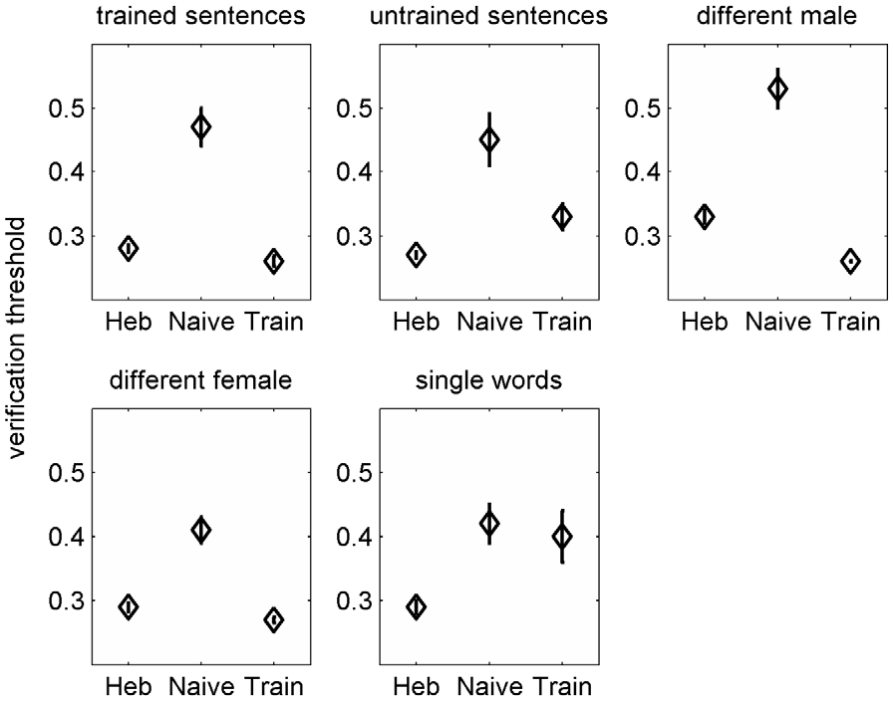
Comparison among naïve native Hebrew speakers, naïve Arabic speakers and trained Arabic speakers. Each panel shows group mean thresholds of (left to right) the Hebrew speakers, the naïve Arabic speakers and the trained Arabic speakers. See text for details.

The Arabic speakers who participated in this study are highly proficient Hebrew speakers. Nevertheless, they are not native speakers, and consistent with previous reports, many of them tend to find spoken Hebrew in their academic environment too rapid. Because it has been suggested that listeners are more negatively affected by naturally fast speech than by artificially compressed speech, it is of interest to compare the performance of trained and untrained (naïve) Arabic speakers on the sentence verification task used in this study to that of native Hebrew speakers with no prior experience with time-compressed speech. To this end mean group thresholds of Hebrew speakers, naïve Arabic speakers (the pre-test data of the control group) and trained Arabic speakers (the post-test data of the trained group) were compared. As shown in Figure 4, naïve Arabic speakers were substantially poorer than naïve Hebrew speakers on all of the stimulus conditions. Planned comparisons revealed that those differences were significant across all conditions (all t_(61)_ < −4, p<0.001). Trained Arabic speakers did not differ from the native Hebrew speakers on the trained condition (t_(61)_ = 0.58, p>0.05) and the untrained female speaker condition (t_(61)_ = 1.00, p>0.05). Furthermore, trained Arabic speakers outperformed the untrained Hebrew speakers on the untrained male condition (t_(61)_ = 2.42, p = 0.019). On the other hand, trained Arabic speakers still had significantly poorer thresholds than the native Hebrew speakers on the conditions to which learning did not generalize (untrained sentences: t_(61)_ = −2.11, p = 0.048; words: t_(61)_ = −2.65, p = 0.015).

## Discussion

Consistent with the hypothesis that learning of time-compressed speech does not end after adaptation to a few dozen sentences, we have shown here that when multi-day training is available, learning can continue through several practice sessions. Furthermore, the pattern of generalization of learning following multiday practice appears qualitatively different from the characteristics of generalization following rapid adaptation to time-compressed speech as reported in previously published works. Rapid adaptation was previously found to be talker and content independent (see Introduction). Likewise, in the current study, participation in the pre/post test sessions only was sufficient to yield improvement (with moderate effect sizes) on three out of the five conditions (the trained condition, untrained sentences and different male). Further training resulted in additional learning, but only of the trained-tokens (whether presented by the trained talker or by different talkers), leading us to suggest that this learning is more specific in nature. Those differential characteristics suggest that similar to the perceptual learning of basic auditory [32], [33], [34] and visual [20], [35] features, learning of time-compressed speech progresses through an initial general phase and a subsequent prolonged and more stimulus-specific phase.

Multiday training lead to the generalization of learning to untrained talkers, and to some (lesser) extent to untrained sentences (as evident by improvement in performance consistency for this measure), but learning did not transfer from sentence-level to word-level verification. A comparison of this pattern of generalization to that described previously for the brief adaptation phase [4], [7], [9], [16] suggests that both stages involve the modification of talker-independent speech representations. On the other hand, consistent with the predictions of the RHT, the learning induced by multiday practice in this study was more stimulus specific than that induced by adaptation to 10–20 sentences. Although this greater specificity could be the result of the repeated presentation of a limited set of stimuli during training rather than due to a real increase in the specificity of learning, we do not think that this is the case, for three reasons. First, a mere increase in the number of tokens did not result in continued learning in earlier studies of adaptation to rapid speech (see Introduction), suggesting that the continued learning in this study was of a different nature. Second, in a series of studies in which speech-in-noise learning was compared between regimens differing in the number of trained tokens (450–2400 individual tokens), no greater generalization to untrained tokens was observed with the larger sets, even though those included fewer repetitions of each individual token (see [36] for a summary). Third, even in studies in which wider generalization was found following practice on larger than on smaller stimulus-sets, stimuli were not unique, but were repeated several times throughout training (e.g., [12], [37], [38]). Another potential explanation for the specificity of learning observed in the current study compared to earlier studies on adaptation to time-compressed speech is differences in task demands. Whereas we used a verification task, in earlier studies listeners were asked to simply report the sentences they heard. Again we do not think that this is the case because we have pilot data showing that asking listeners to repeat the sentences does not change the pattern of generalization. Taken together, we were led to the conclusion that the current pattern of generalization is more consistent with the presence of two different phases of learning.

That learning of time-compressed speech proceeds from an initial rapid phase that generalizes broadly to a subsequent phase that is more stimulus specific suggests that the two phases may engage different forms of learning or modify different types of neural representations of speech. Adaptation to time-compressed speech has been interpreted as a process of attentional weighting during which listeners learn to allocate attention to the cues most relevant for the perception of rapid speech [8]. According to the RHT, attention is allocated by default to high level representations, but subsequent experience makes finer-grained more detailed representations accessible to conscious perception [17]. Therefore, according to the RHT, initial learning should generalize more broadly than subsequent learning which focuses listeners on more specific representations. This account is consistent with the characteristics of the two phases of time-compressed speech learning. It has already been noted that the generalization of adaptation to time-compressed speech is consistent with the RHT [6]. Furthermore, it has been shown that adaptation to time compressed speech engages, in addition to high level auditory areas, premotor areas known to be involved in articulation, suggesting that adaptation might allow the mapping of novel time-compressed stimuli to existing sensory-motor representations [39]. Here, we provide evidence for the remaining RHT prediction that the specificity of learning should increase with prolonged practice. We suggest that with multiday practice on the same set of tokens, the perception of time-compressed speech should start to engage lower level areas in which the representations of speech are rich in spectro-temporal details (as compared to the initially accessible representations) and are not confined to existing articulatory categories (because even the fastest talkers are not likely to have ready articulatory categories corresponding to the compression rates achieved after training).

As for the identity of those representations, the outcomes of the current study suggest the involvement of suprasegmental representations. Otherwise, similar to the cross-language adaptation across languages with similar phonologies [4], learning should have been observed on the lexical decision condition, which was not the case here. Learning of sentences following multiday practice did not generalize to single words, and furthermore, no pre- to post-test improvement on the single words/pseudo-words condition was observed in either group or index of performance (average threshold, performance consistency). This suggests that learning on the time-compressed speech task used here involved longer-term regularities, perhaps relating to suprasegmental characteristics (e.g., the envelope) of the stimuli. A similar interpretation was proposed by Sebastian-Galles and colleagues [9] to account for transfer of learning across languages with shared stress patterns and similar vowel systems (e.g., Spanish and Greek). It has long been recognized that different temporal scales are perceptually relevant. For example, longer-term information about stimulus envelope and periodicity is relevant for the perception of rhythm, stress and intonation [40]. Furthermore, human listeners can learn to comprehend speech in which the fine-grained acoustic representation is severely degraded if the envelope of the original stimulus is maintained (e.g., in users of cochlear implants and in studies in which normal hearing individuals adapt to simulations of cochlear implants [41], [42], [43]). The neural processing of speech involves (at-least) two temporal windows, roughly corresponding to the distinction between segmental and suprasegmental cues, making possible the existence of acoustic representations rich in acoustic information but corresponding to different time constants. Furthermore, the two hemispheres appear differentially sensitive to those time windows (see [44] for a recent review). Taken together, it therefore appears that the idea that learning could involve sublexical yet suprasegmental representations is plausible.

An alternative interpretation for the lack of generalization to the single-word condition is the differences in task demands between the trained condition and the single-word condition. Whereas the training condition required listeners to judge the plausibility of the content of each sentence - a semantic decision, the single-word condition required a lexical one. Therefore, it could be that with prolonged training on the semantic task, listeners became more able to make semantic decisions involving rapid speech, but not lexical decisions. If this is the case, the level of representation modified by training is both semantic and content specific. That is, the semantic representations (which are probably talker independent) of the trained sentences were modified to incorporate their compressed forms. Although we only trained on one task (sentence verification), previous studies indeed show that generalization might depend on task demands, and particularly on the level of processing on which listeners focus during training. For example, listeners trained on talker identification with isolated words, improved their ability to recognize isolated words, whereas listeners who trained on the talker identification task with sentence materials improved their ability to recognize sentences, but not isolated words [45]. Similarly, following practice with vocoded speech, differential patterns of generalization were observed depending on the training task, with wider generalization in a group trained on sentence identification or talker identification than in a group trained on gender identification [46].

Initially, non-native Hebrew speakers had significantly elevated verification thresholds than those of native speakers of Hebrew on all of the conditions used in the study. This finding is consistent with the presence of speech perception difficulties among even highly experienced non-native speakers under non-ideal listening conditions such as the presence of background noise [47], [48], as well as with the reports that highly experienced non-native speakers benefit from slower than average speech rates [2], [10]. Whereas the adaptation potentially induced by participation in the pre-test was not sufficient to bring the performance of the non-native control group to that of naïve native speakers (even in the post-test), after multi-day practice the performance of the trained group was equivalent to that of naïve native speakers on the trained condition as well as on the two untrained conditions to which learning generalized. Taken together, those observations suggest that the long-term learning induced by prolonged experience with a non-native language is more similar in nature to that induced by the rapid adaptation phase. It stands to reason that exposure to language in natural settings does not provide the type of consistent stimulus presentation required to achieve native-like performance. On the other hand, achieving native-like performance on a restricted set of sentences seems rather unuseful. It therefore appears that the practical application of training regimens in this area might require extensive (multi-session) training with a larger stimulus set than that used in the current study. For example, it has been recently reported that among college students (half of which were non-native English speakers), learning during a prolonged training regimen (twenty sessions of approximately 30 minutes each) generalized to untrained speech-in-noise materials [11]. In this study the training materials included multiple degraded-speech passages on different topics.

In summary, we show that non-native speakers improve their ability to perceive time-compressed speech in two phases. A brief adaptation phase and a slower and more stimulus specific phase that follows longer term practice. The presence of those two phases and their characteristics are consistent with the predictions of the RHT. Whether learning in native-speakers follows a similar pattern remains to be determined in further studies. Because the current results suggest constraints on the applicability of training during second language acquisition, further studies designed to understand and overcome those constraints are required prior to the practical application of training regimens in clinical and typical populations. Another issue that requires further investigation is that of optimal training duration. For time-compressed speech, our findings suggest that at the group level, learning asymptotes after two training sessions. Nevertheless, inspection of the individual learning curves shows that for many (about half) of the trained listeners, learning continued beyond that session. Therefore it appears that individual differences in learning rate are an additional factor that has to be considered when attempting to understand the effects of training.
